# Hospital Choice for Cataract Treatments: The Winner Takes Most

**DOI:** 10.15171/ijhpm.2018.77

**Published:** 2018-09-01

**Authors:** Suzanne Ruwaard, Rudy C M H Douven

**Affiliations:** ^1^Netherlands Bureau for Economic Policy Analysis (CPB), Den Haag, The Netherlands.; ^2^Tilburg University (TiU), Tilburg, The Netherlands.; ^3^National Institute for Public Health and the Environment (RIVM), Bilthoven, The Netherlands.; ^4^Erasmus School of Health Policy & Management (ESHPM), Erasmus University, Rotterdam, The Netherlands.

**Keywords:** Hospital Demand, Patient Choice, Quality Indicators, Quality Competition

## Abstract

**Background:** Transparency in quality of care is an increasingly important issue in healthcare. In many international healthcare systems, transparency in quality is crucial for health insurers when purchasing care on behalf of their consumers, for providers to improve the quality of care (if necessary), and for consumers to choose their provider in case treatment is needed. Conscious consumer choices incentivize healthcare providers to deliver better quality of care. This paper studies the impact of quality on patient volume and hospital choice, and more specifically whether high quality providers are able to attract more patients.

**Methods:** The dataset covers the period 2006-2011 and includes all patients who underwent a cataract treatment in the Netherlands. We first estimate the impact of quality on volume using a simple ordinary least squares (OLS), second we use a mixed logit to determine how patients make trade-offs between quality, distance and waiting time in provider choice.

**Results:** At the aggregate-level we find that, a one-point quality increase, on a scale of one to a hundred, raises patient volume for the average hospital by 2-4 percent. This effect is mainly driven by the hospital with the highest quality score: the effect halves after excluding this hospital from the dataset. Also at the individual-level, all else being equal, patients have a stronger preference for the hospital with the highest quality score, and appear indifferent between the remaining hospitals.

**Conclusion:** Our results suggest that the top performing hospital is able to attract significantly more patients than the remaining hospitals. We find some evidence that a small share of consumers may respond to quality differences, thereby contributing to incentives for providers to invest in quality and for insurers to take quality into account in the purchasing strategy.

## Background

Transparency in hospital quality is essential as it contributes to a patients’ ability to make the appropriate hospital choice. In the Netherlands, after the introduction of managed competition in 2006, hospitals started to bargain with health insurers over prices, quality and volume of care. Insurers are likely to be more incentivized to reward quality in their purchasing strategy if consumers indeed take quality into account in their provider choice. A necessary condition for competition to work is that patients respond to quality differences across providers. As a result, better performing hospitals would attract more patients.^1^ Providers are increasingly incentivized to invest in quality thereby contributing to quality of healthcare in the system. The central question of our paper is whether cataract patients take quality into account in hospital choice, and whether high quality hospitals are able to attract more patients.

Evidence suggests that consumers tend to choose better performing providers and are responsive to initiatives that provide quality information.^2^ The decision to visit a hospital may depend on various factors. Besides quality, distance to a hospital, waiting time and information of third parties are also important factors for consumers. In the Netherlands, consumers may retrieve information on quality via the patients’ general practitioner (GP), family and friends and from publicly available quality data.^3^

Several studies have explored the impact of quality on hospital volume (aggregate-level) and or hospital choice (individual-level). Most studies find this impact to be positive but small, and some find a weak or no significant impact.^4-16^ Over time, exploring the impact of quality on hospital choice has become the preferred method.^11^ Moreover, some studies have found nonlinear trends in responding to quality information, these studies tend to find that patients avoid relatively bad hospitals and highly ranked hospitals are not able to attract significantly more patients.^17-19^ This latter finding is counterintuitive. Like in other competitive markets, high-ranked providers that excel and differentiate with respect to quality are expected to attract significantly more patients than their competitors. Our contribution to the literature is that we test this hypothesis by using a quality indicator that measures reputation of hospitals that perform cataract treatments in the Netherlands for the period 2006-2011.

Cataracts are “changes in clarity of the natural lens inside the eye that gradually degrade visual quality.”^20^ Cataracts tend to develop over time, and may lead to vision impairment and blindness.^21,22^ In 2010, cataract was the main cause for blindness worldwide.^21^ Blindness is more common among old age (although it does also exist at younger ages).^22^ Furthermore, visual impairment is more common amongst women than among men.^22^ Cataract is a condition that is fully treatable,^21^ it involves a surgical procedure where the old lens is removed and replaced with a new one.^23^ Moreover, cataract treatments are fairly standard procedures and are associated with low medical risk^ [1] ^. ^24^

In the Netherlands, cataract treatments may be carried out in a hospital or in an independent clinical practice (in Dutch: Zelfstandig Behandelcentra). Historically, practically all Dutch hospitals provided cataract treatments and recently more and more independent treatment centers entered the market. Depending on a patient’s insurance product type, either all providers may be covered (and fully reimbursed), or only contracted providers are reimbursed, and patients are required to pay a (small) share of the costs for non-contracted care providers. At the point of study, there is little selective contracting. Furthermore, patients require a referral from their GP to access medical specialist care, but have freedom with respect to provider choice.

The market for cataract treatments lends itself for this analysis because: (1) quality data at the treatment level is publicly available and reported quality differences are present, (2) cataract treatments are non-emergent and fairly standard procedures, (3) cataract treatments are carried out in practically all Dutch hospitals, and (4) patients have freedom with respect to provider choice.

This paper follows the strategy of Pope^4^ by starting with an aggregate-level analysis followed by an individual level-analysis. The paper starts in section 2 with a brief review of the relevant literature, a description of the dataset and the data analysis is given in the section 3, followed by the individual-level and aggregate-level results in section 4. The paper concludes with a conclusion and discussion in section 5.

## Literature Review

In the health economics literature, the impact of quality has been investigated at the aggregate-level and at the individual-level. At the aggregate-level, studies explore the impact of quality on hospital volume.^4-6,12,13^ At the individual-level, studies investigate the impact of quality on a patients’ hospital choice and relate this to factors such as hospital distance and waiting time.^4,8,9,14-16,18,19,25,26^ One advantage of the individual-level approach over the aggregate-level approach is that it enables estimating how patients make trade-offs between quality and other factors such as distance and waiting times. Quality measures that are used differ across studies.^9^ Studies use either mortality rates,^17^ readmission rates,^8^ patient reported outcomes or report cards^5,6,12-14,18,19^ or, as in our study, hospital reputation and composite scores.^4,15,16^ These studies tend to find that quality has a small but positive impact on either hospital volume or hospital choice, and some find the impact to be weak or insignificant. More research on this topic is warranted because a systematic review in 2011,^27^ concluded that at that time the available evidence was too limited to show how performance data influences the behaviour of consumers, providers and purchasers of healthcare.

Our paper is closely related to Gutacker et al^14^ who argue that hospital choice for a hospital is strongly related to the type of quality that is available. While many studies of quality and choice of hospitals have used general quality measures, such as mortality and readmission rates, few studies use quality indicators for specific treatments. Gutacker et al use detailed patient reports of health outcomes specific for hip replacement, and find that a one standard deviation increase in average health gain increases demand by up to 10%. They also find that more traditional measures of hospital quality are less important in determining hospital choice. In this paper we also use two different types of quality indicators; one indicator for overall hospital quality and one indicator specific for cataract treatments.

Moreover we allow in our estimations for a nonlinear impact of quality information on patient volume or hospital choice. Many previous studies tend to find such effects.^17-19^ For example, Wang et al^18^ finds that after public reporting, surgeons with poor cardiac care report cards treat significantly less patients, while highly ranked surgeons did not treat significantly more patients. Dranove and Sfekas^19^ use a conditional choice model to test whether patients move to better quality hospitals as a result of introducing hospital report cards. They find that patients shift to better performing hospitals after the introduction of hospital report cards. This effect is however mainly driven by patients avoiding lower quality hospitals; they did not find that higher quality hospitals were able to attract significantly more patients.

## Methods

The main dataset consists of all patients who underwent a cataract treatment (as defined by DBC^ [2]^ -code 110005540031) in the Netherlands during the years 2006-2011. This data was obtained from the Dutch Healthcare Authority (NZa)^ [3] ^. For each treatment, the dataset contains the patients’ zip code, the unique hospital code and year of treatment. The total sample consists of 854 613 DBCs (the sum of the total numbers of DBCs for the years 2006-2011, as reported in the first row of Table 1).

**Table 1:** Summary Statistics^a^

	**2006**	**2007**	**2008**	**2009**	**2010**	**2011**
Total DBCs	117 980	139 474	151 972	145 097	151 826	148 264
Total number of hospitals	149	149	151	152	153	153
Average distance (km)	13.48	13.20	13.66	14.26	14.44	16.40
Average waiting time (wk)	6.73	6.71	6.22	5.23	5.25	4.85
Average overall hospital quality (on a scale of 1-10)		5.09	5.14	5.08	5.77	5.49
Average ophthalmologist quality (on a scale of 1-100)			18.48	19.14		

Abbreviation: DBC, Diagnose Behandel Combinatie. ^a^The total number of DBC’s for 2006-2011 was 854 613. In our dataset there are 8 university hospitals, 88 general hospitals in 2006 to 85 in 2011, and the remaining hospitals are independent treatment centers. The average distance to the visited hospital was taken over all 854 613 individuals. The annual average waiting times is taken over all hospitals. Each overall hospital quality score represents the average quality of a hospital for all treatments (and thus not necessarily cataract treatments). Overall hospital quality scores were only available for the years 2007-2011. Each ophthalmologist quality score represents the average quality of ophthalmologists in a hospital. Ophthalmologist quality scores were only available for the years 2008 and 2009. The annual average quality measures are taken over all hospitals. All averages in the table are unweighted averages.

From this we derived the following variables: ‘total DBCs’ (total number of cataract treatments), ‘total number of hospitals,’ (number of hospitals in which cataract treatments were carried out) and ‘average distance (in km)’ (the average distance from the patients zip code to the hospital in km). In addition, we obtained the average waiting times from the NZa, creating the variable ‘average waiting time (in weeks).’ Finally we included two quality variables: one quality score for the specialism ophthalmology, ‘average ophthalmologist quality,’ and a quality score for the overall quality of the hospital ‘average overall hospital quality.’

Table 1 provides the summary statistics on all variables used. Total number of DBCs increase over the years and are carried out in approximately 150 hospitals. The average hospital performs about 1000 cataracts annually. The other variables in the table are discussed in the next section.

### Data description

### Quality

Table 1 summarizes two quality indicators that are obtained from the Dutch weekly magazine Elsevier^28^: “average ophthalmologist quality” and “average overall hospital quality.” Elsevier publishes the quality of Dutch hospitals annually.

**Figure 1:**
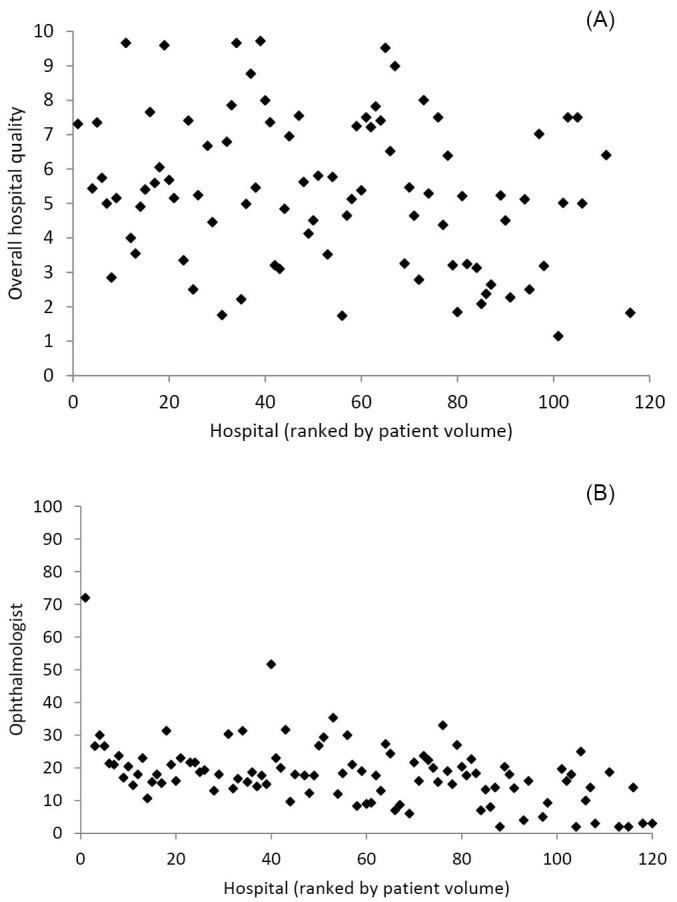
(A) Overall Hospital Quality (Range 0-10). **(B)** Ophthalmologist Quality Scores (Range 0-100).

The indicator ‘average overall hospital quality’ was published in all years: 2007-2011 and is based on process, structure as well as outcomes measures^ [4] ^. During these years Elsevier has changed the specification of their quality indicator. More specifically, in 2007, overall hospital quality was based on seven quality measures, in 2008 on 23, in 2009 on 6, and in 2010-2011 on 4 quality measures. In 2011 the indicators were for example: (1) service/information, (2) shorter waiting times, (3) safety and, (4) effectiveness. These four indicators were based on 183 underlying indicators including for example: usage of ICT, whether an eczema patient is able to contact his dermatologist outside contact hours, whether annual appointments can be scheduled in one day etcetera. In addition, the scaling of the quality measures also differed across the years. For reasons of comparison, the annual overall hospital quality scores were converted to an equal scale of 1-10. Figure 1A shows the average hospital quality scores in the period 2007-2011 per hospital. On the horizontal axis, hospitals are ranked based on patient volume (that is: the lower the patient volume the further to the right). The figure shows a wide variation in quality.

In addition, the Dutch magazine Elsevier published quality indicators for specific specialisms, including ophthalmology. The indicator “average ophthalmologist quality” is based on survey information. Respondents of this survey were asked to judge a maximum of four hospitals, and were asked to indicate whether these hospitals stand out in terms of their medical services and practice management in relation to cataract treatments. The respondents include medical specialists, head nurses and head of departments such as intensive care or operating rooms, GPs, managers and directors of hospitals. Therefore, this indicator is likely to capture the reputation of ophthalmologists in a hospital^ [5] ^. The ophthalmologist quality indicator was only published for the years 2008 and 2009. In 2008 and 2009 there were, 4.787 and 4.441 respondents respectively, of which 2.862 and 2.519 medical specialists. The ophthalmologist average quality scores over the 2 years 2008 and 2009 are between 0-100 and are for each hospital shown in Figure 1B. On the horizontal axis, hospitals are again ranked from high to low patient volume. The figure shows that the largest hospital is an outlier, with a quality score ranging between 70 and 80, and the remaining hospitals have quality scores ranging between about 0-40, with one exception where one medium sized hospital having an average quality score between 50-60.

### Control Variables

The data on yearly average waiting time in weeks per hospital was also obtained from the NZa. The average waiting time was available for the years 2006-2011, the average waiting time of all hospitals in this period was 5 weeks and 6 days (5.80 weeks). Over time, this average waiting time steadily declined, starting from 6 weeks and 5 days (6.73 weeks) in 2006 to 4 weeks and 6 days (4.85 weeks) in 2011 (Table 1). Furthermore, hospitals showed great variation in the average waiting time, the minimum and maximum waiting time in this data set was 0 and 21 weeks.

**Figure 2:**
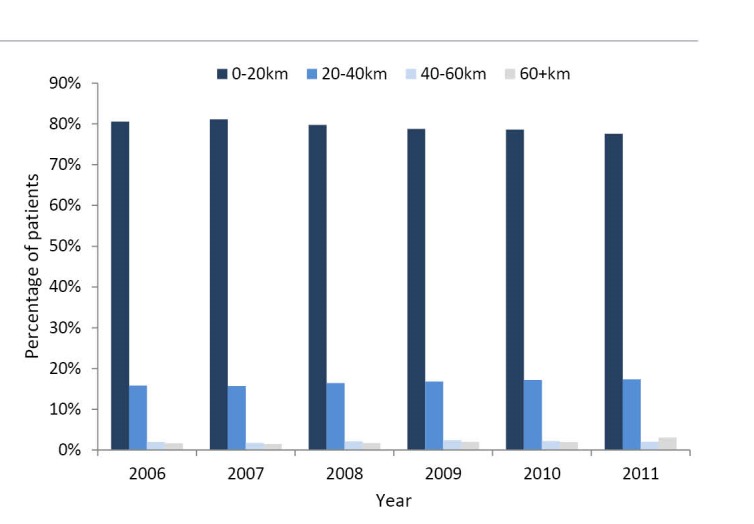
Distance Travelled Over Time (Per Distance Category).

The control variable distance is based on the kilometres between the patients’ zip code of residence and the hospitals’ zip code^ [6] ^. The average number of kilometres that patients travelled to hospitals in this sample increases slightly over time, from 13.48 km in 2006 to 16.40 km in 2011 (Table 1)^ [7] ^. The majority of the patients does not travel far, although there are some outliers. Figure 2 shows the distribution of patients travelling 0-20 km, 20-40 km, 40-60 km and 60+ km. This figure illustrates that approximately 80% of the patients visit a hospital within a range of 20 km, and 15% travels 20-40 km. Very few patients travel to hospitals located further than 40 km. In the total sample, 37% of the patients bypassed the closest hospital, while 88% of the patients who visited the top performing hospital bypassed the closest hospital.

### Estimation Method

### Aggregate-Level Analysis

At the aggregate level, a simple ordinary least squares (OLS) model is used to estimate the effect of quality on patient volume for the years 2008-2011. Similar to Pope^4^ the OLS model is estimated as follows:

*Y*_i,t =_ *α* *+* *β*_1_ *w*_i,t_ *+ β*_2_ *Q*_i,t-1_ *+ Υ*_t_ *+ ε*_i,t_

*Y*_i,t_ represents the total number of cataract treatments carried out at hospital *i*, in year *t*. Variable *w*_i,t_ is the average annual waiting time in weeks for hospital *i* in year* t*. The term *Q*_i,t-1_ is a vector of lagged ophthalmologist and lagged overall quality of hospital I^ [8] ^. The overall hospital quality scores of 2007-2010 were used as lagged quality scores for the years 2008-2011. Since for the ophthalmologist quality scores we do not have information for all years, we used in the regression the quality scores in 2008 as a proxy for the lagged quality indicator for 2008 and the ophthalmologist quality score in 2009 as a proxy for the lagged quality indicator for 2011. Year dummies, *Υ*_t_, are included to capture year-specific effects. Fortunately, the annual variation of hospital quality is limited making these proxies fairly reliable^ [9] ^. To explore to what extent the impact of ophthalmologist quality on patient volume is driven by the top performing hospital, we run the regression one more time without the top performing hospital.

The OLS model has two potential limitations. First, it does not control for differences between hospitals. A fixed effects model is run to control for unobserved provider differences such as: the number of specialists, the resources available, spare capacity.^30^ Second, this OLS model does not allow for the inclusion of the variable distance, therefore the analysis continues with a patient choice model at the individual-level.

### Individual-Level Analysis

A mixed logit model is used for the individual-level analysis, as this is generally used to model hospital choice on a patient-level.^11^ The model is used to analyse how patients make trade-offs between quality, distance and waiting time. The mixed logit model is more flexible than the conditional logit model because it allows for random taste variation, unrestricted substitution patterns and correlation in unobserved factors over time.^31^ Under the mixed logit model the parameters that are associated with each observed variable are not fixed, but allows for variation at the patient level. The conditional logit model on the other hand, assumes the parameters are fixed; hence differences in preferences are related to observed characteristics of the patient and are captured through the inclusion of interaction variables. We use a standard utility function of individual *i* attending hospital *q* in time *t*:

$$U_{i,q,t}=a+{\beta^{\prime }}_{i,t}X_{i,q,t}+e_{i,q,t}$$

*X*_i,q,t_ represents the explanatory variables quality, distance and waiting time. The error term *e*_i,q,t_ is also unobserved and is assumed to be independent and identically distributed. Parameter is a vector of coefficients and is unobserved. Parameter  is treated as a random parameter and is integrated over all its possible values of *β*, then weighted by the density of *β* to obtain the unconditional choice probability, *P*_i,q,t_ of person *i*, choosing hospital *q,* in year *t*.

$$P_{i,q,t}=\int (\frac{e^{\beta^{\prime }x_{i,q,t}}}{\sum_{j}e^{\beta^{\prime }x_{i,q,t}}})*g(\beta )|d\beta$$

Our data set consists of 854 613 patients who are free to choose from 150 hospital locations. Unfortunately, a mixed logit cannot be computed with a data set of this format. Therefore, the dataset is reduced by confining to the years 2009 and 2010, and by restricting the patients’ choice set. The set is reduced to the years 2009 and 2010 because the lagged quality indicator ophthalmologist is only available for these 2 years. In addition, the individual hospital choice set is restricted to the 20 closest hospitals only. So, instead of having all hospitals in the choice set for all patients, we composed a choice set of the 20 closest hospitals only (based on distance) for every individual. Allowing for larger choice sets than the closest 20 hospitals did not alter the results significantly as the majority of the patients’ choices were among the closest 20 hospitals^ [10] ^. These two restrictions resulted for the years 2009 and 2010 in 2 996 205 patient-hospital combinations that are used in the estimations.

To estimate whether people have a stronger preference for the top performing hospital, the model is estimated twice, first with quality for ophthalmology in linear form, and second with quality dummies allowing for nonlinear effects. The average score of the variable quality ophthalmologist in the year 2009 and 2010 is used as the quality indicator. Quality dummies are created for every 10-point interval up until 70-80 (as all scores were <80). Quality dummy 0-10 is excluded to avoid multicollinearity). In both regressions, all coefficients are assumed to be normally distributed.

In addition, the literature states a nonlinear effect of distance where the negative utility of having to travel an additional kilometre is expected to fall with distance. To allow for this nonlinearity, the following distance dummies are incorporated: 0-20 km, 20-40 km, 40-60 km, 60-80 km, 80-100 km and 100+ km (the last dummy variable is again excluded from the regression to avoid multicollinearity).

For reasons of comparison, both regressions were also run using a conditional logit. The conditional logit serves as a good comparison to the mixed logit as this generally yields similar results. The mixed logit is more preferred as it relaxes the independence of irrelevant alternatives assumption and generally yields more precise estimates, the coefficients however tend to be of similar size.^32^

## Results

Table 2 depicts the correlation matrix of the variables at the hospital level: patient volume (total number of DBCs at a hospital), waiting time, ophthalmologist and hospital quality. The matrix shows that both quality variables are positively correlated with patient volume. The variable ophthalmologist quality shows however a much stronger correlation with patient volume than overall hospital quality, 47 and 15 percent respectively. The correlation between the two quality variables is fairly low (12%), indicating that there is a large variation in quality across different specialties in hospitals. Table 3 shows a similar correlation matrix, without the top performing hospital. The correlation of patient volume with ophthalmologist quality almost halves from 48% to 26%. Whereas the correlation of patient volume with hospital quality lowers only slightly from 15% to 13%. In addition, patient volume and waiting time is negatively correlated in both tables which suggests that hospitals with longer waiting times experience somewhat lower patient volumes. Waiting time is also negatively correlated with both quality indicators suggesting that better quality hospitals generally have lower waiting times.

**Table 2:** Correlation Matrix: Volume, Waiting Time, and Quality^a^

	**Patient Volume**	**Waiting Time**	**Ophthalmologist Quality**	**Overall Hospital Quality**
Patient volume	1.0000			
Waiting time	-0.1778	1.0000		
Ophthalmologist quality	0.4748	-0.1434	1.0000	
Overall hospital quality	0.1518	-0.1144	0.1176	1.0000

^a^The coefficients are Pearson correlation coefficients and calculated at the hospital level for all available years (see Table 1). For example, -0.1778 is the Pearson correlation coefficient of patient volume (the annual number of DBC’s in a hospital) correlated with the annual average waiting times for that hospital. The correlation coefficient is calculated for all available years 2006-2011.

**Table 3:** Correlation Matrix: Volume, Waiting Time, and Quality (Excluding the Top Performing Hospital)^a^

	**Patient Volume**	**Waiting Time**	**Ophthalmologist Quality**	**Overall Hospital Quality**
Patient volume	1.0000			
Waiting time	-0.1587	1.0000		
Ophthalmologist quality	0.2568	-0.1153	1.0000	
Overall hospital quality	0.1259	-0.1087	0.0841	1.0000

^a^The coefficients are Pearson correlation coefficients and calculated at the hospital level for all available years (see Table 1). For example, 0.2568 is the Pearson correlation coefficient of patient volume (the annual number of DBC’s in a hospital) correlated with the annual ophthalmologist quality score for that hospital. The correlation coefficient is calculated for the two available years 2008-2009.

### Aggregate-Level Results

Table 4 shows the aggregate level results of the impact of quality on patient volume. The first two columns represent the analysis based on all hospitals, with and without overall hospital quality (columns 2 and 1 respectively). The first two columns show that ophthalmologist quality is positively correlated with patient volume. In the years 2008 and 2009, a one-point increase in ophthalmologist quality (on a scale of 1-100) results in approximately 57 and 64 more patients respectively for the average hospital, which translates into a patient volume increase of 4%. Over time, this impact declined somewhat: in 2010 and 2011, a one-point increase in ophthalmologist quality is associated with a patient volume increase of about 2%. Column 2 shows the results after adding overall hospital quality to the regression. The results show that overall hospital quality is insignificant. Overall hospital quality does also not appear to affect the results for ophthalmologist quality, as the impact of ophthalmologist on patient volume is similar in columns 1 and 2. The remaining indicator, waiting time, is significant and negatively associated with patient volume: an increase in waiting time by one-week results in 66 fewer patients, or a patient volume decline of 4 percent for the average hospital.

**Table 4:** Patient Volume, Ophthalmologist Quality and Overall Hospital Quality

	**All Hospitals**		**Without Top Performing Hospital**	
	**(1)**	**(2)**	**(3)**	**(4)**
Ophthalmologist quality 2008	56.56*** (10.35)	52.96*** (10.84)	33.76** (11.23)	27.09* (11.64)
Ophthalmologist quality 2009	63.93*** (11.44)	61.43*** (11.73)	31.90* (12.97)	27.95* (13.09)
Ophthalmologist quality 2010	34.08*** (9.08)	35.55*** (9.64)	15.67 (10.74)	14.40 (11.45)
Ophthalmologist quality 2011	34.07*** (9.21)	32.77** (9.73)	5.69 (11.03)	1.06 (11.61)
Overall hospital quality 2008		18.40 (38.28)		13.85 (34.73)
Overall hospital quality 2009		43.62 (39.71)		39.49 (36.02)
Overall hospital quality 2010		-47.56 (59.58)		-48.57 (54.04)
Overall hospital quality 2011		25.61 (44.88)		38.94 (40.81)
Waiting time	-66.11*** (15.24)	-66.75*** (16.96)	-64.60*** (14.11)	-65.68*** (15.12)
N	317	284	313	280
Adjusted R2	0.27	0.26	0.10	0.08

(1) Year dummies are not presented here. (2) * Significant at *P* < .05; ** Significant at *P* < .01; *** Significant at *P* < .001. (3) The sample size in column (2), N = 284, and (4), N = 280, is smaller than in column (1), N = 317, and (3), N=313, because for 33 hospitals the overall hospital quality indicator was missing.

The same analysis was then done after omitting the top performing hospital in the regression. The results are shown in columns 3 and 4. Comparing columns 1 and 2 with columns 3 and 4 indicates that the positive impact of quality on patient volume is largely driven by the top performing hospital. After eliminating the top performing hospital from the sample, the impact of ophthalmologist quality on patient volume halves and is no longer significant in 2010 and 2011. In 2008 and 2009, a one-point increase in ophthalmologist quality now results in respectively 27 and 28 more patients, which translates into a patient volume increase of about 2% (instead of 4%).

In addition to the OLS model, a fixed effects model (not reported here) was also run using the same variables. In the fixed effects model however none of the variables turn out to be significant^ [11] ^.

### Individual-Level Results

The individual level results are presented in Table 5. For every variable the mean and standard error of the coefficient is presented in the first row. The second row shows the value of the standard deviation of the coefficient and the corresponding standard error.

**Table 5:** Mixed Logit

**Parameter**		**(1)**		**(2)**	
		**Value**	**SE**	**Value**	**SE**
Ophthalmologist quality	Mean of coefficient	0.032***	(0.00)		
	SD of coefficient	0.01***	(0.00)		
Ophthalmologist quality 70-80	Mean of coefficient			2.53***	(0.02)
	SD of coefficient			0.70***	(0.05)
Ophthalmologist quality 50-60	Mean of coefficient			0.80***	(0.04)
	SD of coefficient			0.20	(0.13)
Ophthalmologist quality 30-40	Mean of coefficient			0.90***	(0.02)
	SD of coefficient			0.96***	(0.03)
Ophthalmologist quality 20-30	Mean of coefficient			1.09***	(0.01)
	SD of coefficient			0.01	(0.02)
Ophthalmologist quality 10-20	Mean of coefficient			0.87***	(0.01)
	SD of coefficient			0.01	(0.01)
Distance: 0-20 km	Mean of coefficient	7.50***	(0.06)	7.54***	(0.06)
	SD of coefficient	0.11**	(0.04)	0.00	(0.01)
Distance: 20-40 km	Mean of coefficient	4.53***	(0.05)	4.55***	(0.06)
	SD of coefficient	0.02	(0.02)	0.00	(0.01)
Distance: 40-60 km	Mean of coefficient	2.22***	(0.06)	2.26***	(0.06)
	SD of coefficient	0.18	(0.10)	0.05	(0.03)
Distance: 60-80 km	Mean of coefficient	1.19***	(0.06)	1.25***	(0.06)
	SD of coefficient	0.07	(0.06)	0.02	(0.05)
Waiting time	Mean of coefficient	-0.01***	(0.00)	-0.02***	(0.00)
	SD of coefficient	-0.00	(0.00)	0.00	(0.00)
Log likelihood		-242 184		-240 920	
No. of observations		2 665 880		2 665 880	

Abbreviations: SE, standard error; SD, standard deviation. (1) Year dummies are not presented here. (2) * Significant at *P* < .05; ** Significant at *P* < .01; *** Significant at *P* < .001.

Table 5, column 1, confirms the aggregate level findings in that people prefer quality hospitals and hospitals that are associated with lower waiting times. The indicator on ophthalmologist quality is positive and statistically significant (0.032). The mean coefficient on waiting time is negative and statistically significant (-0.01). Furthermore, the individual level results suggest that patients prefer hospitals that are close by and that the impact of distance on hospital choice is nonlinear. The mean coefficient on the distance dummies grows smaller as distance grows: for example, people prefer a hospital within a range of 0-20 km as opposed to 20-40 km, as the corresponding mean coefficients are 7.50 and 4.53 respectively. Furthermore, the gap between the coefficients becomes smaller as distance grows: 7.50-4.53>4.53-2.22>2.22-1.19, implying that the negative utility derived from having to travel an additional kilometre declines with distance.

Although the indicators quality and distance cannot be interpreted individually, the willingness to travel for quality can be estimated. This is done by comparing the utility derived from quality and distance: the coefficient on ophthalmologist quality is .032, and the difference between the utility gained from choosing a hospital within a range of 0-20 km as opposed to 20-40 km is 2.97 (7.50-4.53 = 2.97). This suggests that patients value the hospital being within a range of 0-20 km as opposed to 20-40 km 9 times more than a hospital having 10 points higher quality. In other words, for every 10 patients, one patient will choose to travel 20-40 km for a 10-point quality gain, while the other nine will visit a hospital that is within 20 km. More specifically, the results also allow us to calculate by how many kilometres patients are willing to travel for a one-point increase in quality. For example, if a hospital is located within a range of 20 km, the negative utility for travelling an additional kilometre is 2.97/20 = 0.15. This implies that patients are willing to travel 0.032/0.15 = 0.2 km more for a one-point increase in quality.

Table 5 column 2 suggests that people have a strong preference for the top performing hospital, but appear indifferent between the remaining hospitals. The coefficient mean on ophthalmologist 70-80 (2.53) is more than two times higher than that of the remaining quality dummies, suggesting that patients have a stronger preference for the top performing hospital. Given that the coefficient means on the remaining quality dummies are similar (0.80: 0.90: 1.09: 0.87), patients appear indifferent between these hospitals. Hospitals falling in these categories are however preferred over hospitals that fall in the reference group (Ophthalmologist: 0-10).

Patients show moderate variation in how they make trade-offs when choosing a hospital. For some of the variables the standard deviation of the coefficient is significant, which implies that there is patient heterogeneity. More specifically, column 2 shows that patient heterogeneity exists for hospitals with a quality score ranging between 70-80 and 30-40. For the remaining quality categories, patient heterogeneity is not significant. In addition, we find that patients do not vary significantly in how they value distance (except for distance dummy 0-20 km in the first regression).

As a robustness check, the results of the conditional logit are presented in Table S1 in Supplementary file 1. The table shows almost identical results to the mixed logit estimates.

## Discussion and Conclusion

This paper explores trends in quality on hospital volume and hospital choice. Both the aggregate-level and individual-level results suggest that our ophthalmologist quality measure is positive correlated with hospital volume and hospital choice, similar to previous studies.^4-6,8,11,14,15^ As in Gutacker et al^14^ we find only a significant result for the ophthalmologist quality indicator and not for the overall hospital quality indicator. Moreover, the positive impact is non-linear, where the top performing hospital attracts significantly more patients than would be expected based on linear quality differences. In contrast, some previous studies found a nonlinear impact in that after reporting on quality, patient volume for poor performing providers declined, whereas patient volume did not increase for high performing hospital.^7,17-19^ These findings may be related to the type of treatment and quality variable considered. For example, Cutler et al,^17^ Dranove and Sfekas,^19^ and Wang et al^18^ all relied on mortality rates for cardiac care. As the chance and severity of complications for such a complex treatment are likely to be higher than for relatively simply cataract treatments, people may be more sensitive to quality differences in the bottom segment of the market and respond by avoiding poor performers. Mennemeyer et al^7^ show that information about mortality rates that were different from the patients expectations did not alter market shares significantly; however reporting on unexpected deaths in the news had a significant negative impact on market shares. The negative information on unexpected deaths may be driving patients away, which is again reflected in the bottom segment of the market only. In addition, the distribution of the quality variable may be different in other previous studies. Our results suggest a strong impact at the top segment of the hospital market because the dataset contains a clear outlier in the upper segment, whereas the distribution of datasets used in other studies may not contain such an outlier at the top segment.

Furthermore, the findings suggest that there is moderate variation in how patients make trade-offs. Patients tend to either value quality and visit the top performing hospital, or they visit the nearest hospital. Although the top performing hospital has attracted a lot of people from far away, over the years far away patients seem to be increasingly travelling to other hospitals as well. We cannot explain this new trend in the market with our limited dataset but it suggest that other aspects, such as marketing, new quality measures that increasingly become available^ [12]^ or that enhanced competition plays a role. For example, the liberalization of the Dutch hospital market has led to more specialization of ophthalmology hospitals and new independent treatment centers have entered the market.

## Limitations

This research has several limitations. Most importantly, we considered only two imperfect indicators to measure hospital quality. Future research, with better quality data, will be necessary to see whether our results are robust. Healthcare quality has many different dimensions and in many countries more and more efforts are undertaken to measure quality. However this process is going slowly and for many hospital treatments decent output quality indicators are still not available. This research incorporated two quality indicators, one measure for overall hospital quality, and one specialism specific quality indicator. The two indicators reflect only imperfectly the underlying quality of a hospital and may also reflect hospital reputation. However, we used these quality indicators because they were available to the public. Some patients may have actually used this information to choose a hospital while others may have obtained a referral from their GP. Therefore, other hospital attributes (eg, reputation) or other sources of quality information (eg, GPs) that are correlated with these quality indicators may also explain our results. Unfortunately, the specialisms specific quality indicator for ophthalmology was only available for 2009 and 2010. Therefore, in our individual-level analyses we could only use these two years and in our aggregate-level analyses proxies were used for the remaining years, thereby weakening the possibility to interpret the impact of quality on volume and patient choice.

In addition, with respect to the ophthalmologist quality indicators, the top performing hospital is a clear outlier, largely driving the results. This allows for a careful interpretation of our results which may be very market specific. Also, the quality variable is imperfect because it is based on a survey and probably incorporates other quality aspects besides reputation, such as outcome, process and structural quality indicators. Since the survey is based on the opinions of GPs and other medical specialists, the indicator may also be positively correlated with referral patterns because survey respondents may provide referrals themselves. Another limitation of this variable is however that differences in quality scores are difficult to interpret; the cataract treatments are relatively easy to perform, so differences in medical outcomes are likely to be small. However, the fact that the top performing hospital is a clear outlier is confirmed by their reputation; it has acquired the status of centre of excellence and has received several additional awards for its quality in the past year.

Furthermore, owing to the fact that the dataset was too large for conducting a mixed logit model we were forced to make decisions with respect to reducing the sample size. We restricted the choice set to the 20 closest hospitals for every person. Allowing for larger choice sets did not alter the results significantly.

Whether a winner takes most strategy is applicable to other treatments will probably depend on the nature of the treatment and specific market characteristics. The findings suggest that for relatively standard cataract treatments it is possible to become a dominant player in the market. However, for some treatments a winner takes all strategy may be less rewarding. For example, in the market for kidney transplants the availability of transplants is very important and in case of urgent care the accessibility of care may be one of the most important factors. On the other hand, a winner takes most strategy may be rewarding for chronic diseases, as these patients may be more sensitive to quality differences. Further research would be needed to explore whether “a winner takes most” occurs for other treatments as well.

## Ethical issues

We obtained the cataract data from the Dutch Healthcare Authority. This data is proprietary and not publicly available. The quality data is publicly available and can be obtained from the authors upon request.

## Competing interests

Authors declare that they have no competing interests.

## Authors’ contributions

Both authors were involved in the study design and analysis of the work. SR drafted the manuscript and RD contributed to revisions of the manuscript.

## Authors’ affiliations

^1^Netherlands Bureau for Economic Policy Analysis (CPB), Den Haag, The Netherlands. ^2^Tilburg University (TiU), Tilburg, The Netherlands. ^3^National Institute for Public Health and the Environment (RIVM), Bilthoven, The Netherlands. ^4^Erasmus School of Health Policy & Management (ESHPM), Erasmus University, Rotterdam, The Netherlands.

## Endnotes


[1] In general, reverse causality may be a problem in these studies, where volume may be affecting quality due to learning effects. This study tries to rule this reverse causality argument out by focussing exclusively on standard day-cataract treatments, and thus removing the more complex cataract treatment from our sample. Our communication with ophthalmologists confirmed that the cataract treatments that we study in this paper are relatively straightforward and could be performed by all eye-specialists. The reverse causality argument is much more relevant for highly complex care, such as treating heart attacks where specialization is important.

[2] In the Netherlands, hospital products are classified according to the Diagnosis Treatment Combination system (in Dutch: Diagnose Behandel Combinatie, DBC). It is a classification system similar to the Diagnosis Related Groups (DRGs) in the United States.

[3] The dataset includes all Dutch hospitals and almost all independent treatment centers that perform cataract treatments. We might be missing a few independent treatment centers as these were not legally required to report their DBCs to the Dutch Healthcare Authority.

[4] A process indicator relates to the activities and tasks the provider executed in delivering care, structure indicators relate to the number of resources and type of resources used in delivering care, and outcome measures relate to the impact the process of care has on health.^29^

[5] When Elsevier published a new quality indicator for eye specialism in 2011, it was no longer based on surveys, but it was based on publicly available performance indicators,^28^ the top-performing hospital in our study was not among the seven best hospitals in 2011.

[6] Another possible way to estimate travelling would have been in terms of time required to travel to the hospital. For example, Varkevisser et al^25^ use travel time by car between the patient’s zip code and hospital zip code. Actual travel time depends on the route and accessibility of public transportation. However, given that the Netherlands is a very densely populated country with good infrastructure, the distance in kilometres will be not that different from distance in travelling time, especially for people travelling far.

[7] For the hospital with highest ophthalmologist score for quality, patients were willing to travel further, namely 21.10 km.

[8] Some studies insert the absolute values of quality (Bundorf et al,^5^ Jung et al,^9^ Varkevisser et al^8^), other studies use rankings (Pope^4^).

[9] Indeed, we find a strong correlation of 72% for lagged ophthalmologist quality scores in the years 2009 and 2010.

[10] Sivey^26^ and Howard^13^ use a similar approach, they restrict the choice set to the closest 10 hospitals only. Our choice set is somewhat larger because the focus of this study is on the top performing hospital, and for precisely this hospital patients are willing to travel far. If a patients’ choice falls outside his/her choice set, the mixed logit is not able to estimate someone’s preferences hence the patient is dropped from the sample. If we allow for a choice set of twenty hospitals, only 2% of the patients are eliminated, and, only 7% of the patients that visited the top performing hospital is dropped. If the choice set is restricted to the closest ten hospitals only, then 5% of the patients are eliminated from the sample of which 18% of the patients travelled to the top performing hospital.

[11] Results are available upon request. The fixed effects model yields insignificant outcomes as quality variation over time tends to be small, hence quality is largely absorbed in the fixed effects.

[12] The fact Elsevier changed the quality indicator may have had a (negative) impact on the market position of the top performing hospital. In addition, in the last years an increasing number of other quality indicators have become available. For instance, the Dutch website www.kiesbeter.nl collects and publishes quality indicators. These indicators are process indicators such as how does a patient value the communication with an ophthalmologist, a nurse and communication about medication. We tested also whether patient choice was related to these process indicators. Although not reported here, we found for two process indicators only a very weak positive correlation while the third, communication with an ophthalmologist, was weakly and negatively correlated with hospital choice. During the years of our study, these indicators received less attention in the public media. In addition, these indicators do not necessarily reflect outcome quality of a treatment.


## Key messages

**Implications for policy makers**

- Quality competition among hospitals might be possible to a certain degree.
- Only top performing hospitals attract patients from all over the country.
- Most patients choose a hospital in their own region.
- Patients do not seem to respond to all types of quality indicators. Patients seem to be more responsive to outcome indicators than to process

**Implications for public**

Conscious consumer choices may incentivize healthcare providers to deliver better quality of care. This paper studies the impact of quality on hospital volume and hospital choice for cataract treatments in the Netherlands. We find that the top performing hospital is able to attract significantly more patients than the remaining hospitals.

## Supplementary files

Supplementary file 1 contains Table S1.
