# Supplementary material for: Hospital Choice for Cataract Treatments: The Winner Takes Most
Source: Int J Health Policy Manag. 2018 Sep 1;7(12):1120–9. doi: 10.15171/ijhpm.2018.77 (PMC6358653; doi:10.15171/ijhpm.2018.77)
Supplement: Supplementary file 1 — contains Table S1. [file ijhpm-7-1120-s001.pdf]

Supplementary file 1

Table S1. Conditional Logit

	(1)	(2)
**Ophthalmologist**	.032*** (.000)	
**Ophthalmologist 70-80**		2.57*** (.02)
**Ophthalmologist 50-60**		.81*** (.04)
**Ophthalmologist 30-40**		1.03*** (.02)
**Ophthalmologist 20-30**		1.10*** (.01)
**Ophthalmologist 10-20**		.88*** (.01)
**Distance**		
**0-20 km**	7.47*** (.06)	7.42*** (.06)
**20-40 km**	4.51*** (.06)	4.47*** (.06)
**40-60 km**	2.22*** (.06)	2.22*** (.06)
**60-80 km**	1.18*** (.06)	1.21*** (.06)
**Waiting time**	-.01*** (.001)	-.01*** (.001)
**Log likelihood**	-242,203	-241,138
**# of observations**	2,665,880	2,665,880

1.) Year dummies are not presented here.

2.) * significant at $P < .05$; ** significant at $P < .01$; ***significant at $P <.001$
